# Off-label use of biological agents in the management of uveitis: experience from a pediatric rheumatology centre

**DOI:** 10.1186/1546-0096-9-S1-P64

**Published:** 2011-09-14

**Authors:** Raquel Campanilho-Marques, Ana Filipa Mourão, Sónia Melo Gomes, João Eurico Fonseca, Helena Canhão, Carla Macieira, Filipa Ramos, JA Pereira da Silva

## Background

Uveitis is a common extra-articular feature in juvenile idiopathic arthritis (JIA), particularly in female patients with the extended oligoarticular subtype. A significant number of patients already have ocular complications at time of diagnosis of uveitis. JIA-associated uveitis carries significant ocular morbidity that can progress into adulthood. Treatment with immunosuppressive agents can reduce the risk of ocular complications. Although controlled clinical trials are under way to assess the efficacy of biologic agents in JIA-associated uveitis, therapies with these agents seem to have encouraging results.

## Aims

To evaluate the efficacy of the treatment of refractory uveitis with tumor necrosis factor antagonist (TNF) in a Pediatric Rheumatology Centre.

## Methods

We reviewed the case notes of patients followed in our outpatient clinic, diagnosed with uveitis that are being actively treated with biological agents.

## Results

Five patients: four had JIA (two with the seronegative polyarticular subtype, one persistent oligoarticular, one systemic JIA) and one had chronic idiopathic uveitis. The mean age of these patients was 12.6±7.4 years old, the mean disease duration was of 4.8±1.1 years and the mean disease duration at the beginning of the biological treatment was of 2.2±1.6 years. All the patients were treated with anti-TNF agents with a mean duration of 26.4±19.2 months: three patients with infliximab and two with etanercept. Only one child was switched from etanercept to infliximab due to therapy failure. The concomitant immunosuppressive therapy in all the patients was reduced and only one patient remained on additional prednisolone (10 mg/day). No serious adverse events were registered.

## Conclusion

Our case series provides evidence that successful outcomes may be achieved with biological immunosuppressive therapy, although clinical trials are required to define the real efficacy of this strategy.

